# Comparison of bacterial genome assembly software for MinION data and their applicability to medical microbiology

**DOI:** 10.1099/mgen.0.000085

**Published:** 2016-09-08

**Authors:** Kim Judge, Martin Hunt, Sandra Reuter, Alan Tracey, Michael A. Quail, Julian Parkhill, Sharon J. Peacock

**Affiliations:** ^1^​ Department of Medicine, University of Cambridge, Level 5, Addenbrookes Hospital, CB2 0QQ Cambridge, UK; ^2^​ Wellcome Trust Sanger Institute, Wellcome Trust Genome Campus, CB10 1SA Hinxton, UK; ^3^​ London School of Hygiene and Tropical Medicine, University of London, Keppel St, WC1E 7HT London, UK

**Keywords:** MinION, antimicrobial resistance, assembly, software, long reads, plasmid, antimicrobial resistance, assembly, software, long reads

## Abstract

Translating the Oxford Nanopore MinION sequencing technology into medical microbiology requires on-going analysis that keeps pace with technological improvements to the instrument and release of associated analysis software. Here, we use a multidrug-resistant *Enterobacter kobei* isolate as a model organism to compare open source software for the assembly of genome data, and relate this to the time taken to generate actionable information. Three software tools (PBcR, Canu and miniasm) were used to assemble MinION data and a fourth (SPAdes) was used to combine MinION and Illumina data to produce a hybrid assembly. All four had a similar number of contigs and were more contiguous than the assembly using Illumina data alone, with SPAdes producing a single chromosomal contig. Evaluation of the four assemblies to represent the genome structure revealed a single large inversion in the SPAdes assembly, which also incorrectly integrated a plasmid into the chromosomal contig. Almost 50 %, 80 % and 90 % of MinION pass reads were generated in the first 6, 9 and 12 h, respectively. Using data from the first 6 h alone led to a less accurate, fragmented assembly, but data from the first 9 or 12 h generated similar assemblies to that from 48 h sequencing. Assemblies were generated in 2 h using Canu, indicating that going from isolate to assembled data is possible in less than 48 h. MinION data identified that genes responsible for resistance were carried by two plasmids encoding resistance to carbapenem and to sulphonamides, rifampicin and aminoglycosides, respectively.

## Data Summary

1. Sequencing data has been deposited in the ENA under the accession numbers ERS634378: ERR1341575 (MinION pass reads) ERR1341574 (MinION fail reads) and ERR885455 (Illumina reads)
  (url – http://www.ebi.ac.uk/ena/data/view/ERS634378)
2. Supporting data, including assemblies, fast52fastq.py script and QUAST output has been made available through a Github repository
  (url - https://github.com/kim-judge/minionassembly)
3. Manually finished genome has been deposited in ENA accession number: FKLS01000001-FKLS01000010
  (url http://www.ebi.ac.uk/ena/data/view/FKLS01000001)

## Impact Statement

The Oxford Nanopore MinION sequencing technology has several advantages for pathogen sequencing in medical microbiology, but ongoing analysis needs to keep abreast of technological improvements to the instrument and release of new analysis software. Clinical use also requires the generation of data in a timeframe that can inform medical decisions. We compared the performance of four open-access software tools in assembling genome data generated by MinION for a multidrug-resistant isolate of *Enterobacter kobei*. We determined the optimal software in terms of accuracy and speed, and showed how sequence data can be used as early as 9 h into the sequencing run to generate assembled whole genomes. Sequence data detected the genes responsible for resistance to numerous clinically important antibiotics, and assemblies allowed these to be assigned to one of two mobile elements (plasmids). Our findings are relevant to biotechnologists working in medical practice, and to those working in the field of molecular epidemiology who study mobile elements that spread antimicrobial resistance within and between bacterial species of medical importance. Entire bacterial genomes can now be assembled without access to compute clusters or expensive sequencing hardware.

## Introduction

The Oxford Nanopore MinION is a commercially available long read sequencer that connects to a personal computer through a USB port. It is able to generate relatively small amounts of data, making it ideally suited to working with microbes such as bacteria and viruses. To date, the technology has shown promise for microbiological applications, including the delineation of position and structure of bacterial antibiotic-resistance islands (Ashton *et al.*, 2014), assembly of bacterial genomes (Loman *et al.*, 2015; Risse *et al.*, 2015) and tracking of viral outbreaks (Quick *et al.*, 2016; Zika Real time Sequencing Consortium, 2016). This has been supported by the development of analysis tools for MinION data.

MinION data has been shown to be of sufficient quality to accurately detect the presence of antimicrobial-resistance genes (Bradley *et al.*, 2015; Judge *et al.*, 2015; Cao *et al.*, 2015), but these studies focused on mapping long-read data to an existing reference to detect them. Here, we evaluate the performance of four open-access software tools in creating *de-novo* assemblies of genomic data, including plasmids, for a multidrug-resistant isolate of *Enterobacter kobei*. We consider factors key to medical microbiology including accuracy, time taken to generate assemblies and whether the assemblies were of sufficient quality to provide information on the presence and structure of plasmids carrying clinically relevant antimicrobial-resistance genes.

## Methods

### Microbiology.

A multidrug-resistant *E. kobei* isolate was cultured from untreated wastewater in the United Kingdom in 2015 (unpublished data). A freezer vial was prepared based on a single colony, maintained at −80 °C and re-grown from frozen stock for antimicrobial susceptibility testing and DNA extraction. Susceptibility testing was performed using the N206 card on the Vitek 2 instrument (bioMérieux) calibrated against European Committee on Antimicrobial Susceptibility Testing (EUCAST) breakpoints.

### Illumina sequencing and bioinformatic analyses.

DNA extraction and library preparation was performed as previously described (Quail *et al.*, 2012). In brief, 0.5 µg DNA was sheared and end-prepped, A-tailed and adapter ligated according to the Illumima protocol. The library was amplified with six cycles of PCR using Kapa HiFi 2× mastermix (KK2601, Kapa Biosystems). The mean insert size of the library was approximately 200 bp. DNA libraries were sequenced using the HiSeq platform (Illumina) to generate 100 bp paired-end reads. Reads were trimmed using Trimmomatic (Bolger *et al.*, 2014) to remove adapter sequences and regions of low quality and overlapping reads were merged using PEAR (Zhang *et al.*, 2014), with the reverse reads reverse complemented using fastaq. *De novo* assemblies were generated using Velvet (Zerbino & Birney, 2008) to create several assemblies by varying the kmer size. The assembly with the best length for which 50 % of all bases in the sequences are in a sequence of length L (N50) was chosen and contigs smaller than 300 bases were removed. The scaffolding software SSPACE was employed (Boetzer *et al.,* 2010) and assemblies further improved using 120 iterations of GapFiller (Boetzer & Pirovano, 2012). Species identification was based on analysis of *hsp60* and *rpoB*, as previously described (Hoffmann & Roggenkamp, 2003). To detect acquired genes encoding antimicrobial resistance, the *de-novo* assembly was compared by BLAST to a manually curated version of the ResFinder database (compiled in 2012) (Zankari *et al.*, 2012) as described previously (Reuter *et al.*, 2013).

### MinION sequencing and bioinformatic analysis.

DNA was extracted using the QiaAMP DNA Mini kit (Qiagen), and quantified using the Qubit fluorimeter (Life Technologies). Sample preparation was carried out using the Genomic DNA Sequencing Kit SQK-MAP-006 (Oxford Nanopore Technologies) following the manufacturers instructions, including the optional NEBNext FFPE DNA repair step (NEB). A 6 µl aliquot of pre-sequencing mix was combined with 4 µl Fuel Mix (Oxford Nanopore), 75 µl running buffer (Oxford Nanopore) and 66 µl water and added to the flow cell. The 48 h genomic DNA sequencing script was run in MinKNOW V0.50.2.15 using the 006 workflow. Metrichor V2.33.1 was used for base calling. The flow cell was reloaded at 24 h with the pre-sequencing mix prepared as above. MinION and Illumina sequence data have been deposited in the European Nucleotide Archive (Data citation 1).

Basecalled MinION reads were converted from FAST5 to FASTQ formats using the Python script fast52fastq.py. Read mapping was carried out to assess the quality of data and coverage using the BWA-MEM algorithm of BWA v0.7.12 with the flag –x ont2d (Li, 2013). Output SAM files from BWA-MEM were converted to sorted BAM files using SAMtools v0.1.19-44428cd (Li *et al.*, 2009). Assembly using MinION data only was undertaken using PBcR (Koren *et al.*, 2012), Canu (Berlin *et al.*, 2015) and miniasm (Li, 2016). Canu version 1.0 was run using the commands maxThreads=8 maxMemory=16 useGrid=0 nanopore-raw. The PBcR pipeline with CA version 8.3rc2 was run using the options length 500, partitions 200 and the spec file shown in Supplementary Text 1, available in the online Supplementary Material. Minimap and miniasm were run as specified (Li, 2016). The resulting assembly was polished using Nanopolish v0.4.0 with settings as specified (Loman *et al.*, 2015), with Poretools (Loman & Quinlan, 2014) used to extract fasta sequences from fast5 files in the format required by nanopolish using the option fasta. Hybrid assemblies were generated using SPAdes 3.8.1 (Bankevich *et al.*, 2012) using the option ‘–careful’, then filtered to exclude contigs of less than 1 kb. All assemblies were assessed against the manually finished assembly using QUAST (Gurevich *et al.*, 2013) version 3.2 (Table S1, available in the online Supplementary Material). Assemblies were annotated using Prokka (Seemann, 2014). Figures were generated using multi_act_cartoon.py (Git Hub, 2016) and MUMmer (Kurtz *et al.,* 2004) version 3.23. Assemblies and scripts are available online (Data citation 2).

**Table 1.** Comparison of assembly software: number and size of contigs, errors and time/memory requirements

Assembly	PBcR	Canu	Miniasm	Miniasm & Nanopolish	SPAdes	Illumina	Manually finished
Number of contigs	21	15	16	16	13	90	10
Number of bases	5490929	5542520	5843777	5673354	5576147	5454767	5586413
Largest contig (bases)	1615977	2782732	1548218	1504104	5303011	686305	5031167
Mean contig (bases)	261473	369501	365236	354585	428934	60608	620713
N50*	1197808	2782732	661959	641515	5303011	153115	5031167
Total mis-assemblies	5	2	0 (analysis failed)	3	5	6	na
Mismatches per kb	1.0038	0.3494	6.6578	5.4843	0.0371	0.0355	na
Indels per kb	12.1668	7.769	18.6418	8.987	0.0353	0.0322	na
Memory requirement	7 GB	8 GB	3 GB	3 GB & 4 GB	2 GB	4 GB	na
Run time	8 h	2 h	2 min	2 min & 3 days 11 h	3 h	3 h	na
Total CPU time†	79728	54745	124	9450274	9164	12514	na
Number of threads	16	8	2	2 & 16	16	2	na

*N50: a weighted median statistic. Half (50 %) of the assembly is contained in contigs greater than or equal to a contig of this size.

†Total CPU (Central Processing Unit) time: The amount of time used by the CPUs actively processing instructions. Run time, or ‘real’ time, may be longer, as it includes idle time or time spent waiting for input or output, or may be shorter if the workload is shared between more than one CPU.

### Manually finished genome.

Assemblies were generated using Canu and SPAdes, as before. A gap5 database was made using corrected MinION pass reads from the Canu pipeline and Illumina reads. Manual finishing was undertaken using gap5 (Bonfield & Whitwham, 2010) version 1.2.14 (Fig. S1), giving one chromosome and eleven confirmed plasmids. Icorn2 (Otto *et al.*, 2010) was run on this for five iterations. The start positions of the chromosome and plasmids were fixed using circlator (Hunt *et al.*, 2015) 1.2.0 using the command circlator fixstart. This assembly was annotated using Prokka (Seemann, 2014). Where the Canu and SPAdes assemblies did not match with regards to suspected integration of a plasmid into the chromosome, this was additionally investigated using long-range PCR. The assembly and annotation is available online (Data citation 3).

## Results

Our analyses were based on a multidrug-resistant *E. kobei* isolate cultured from sewage. This was selected as a model organism on the basis of its multidrug-resistant phenotype (including resistance to the carbapenem drugs), and because of the additional challenge of working with an organism for which there was no available assembled whole-genome sequence and so reflecting a real-life scenario.

Raw data on the *E. kobei* genome from a single flow cell was initially analysed using the Oxford Nanopore base calling software and defined as pass or fail based on a threshold set at approximately 85 % accuracy (Q9) and including only 2D reads, where data is generated from both the forward and reverse strand of DNA as it passes through the nanopore. The error rate of MinION pass data exceeded that of the Illumina data (0.048 insertions, 0.027 deletions and 0.089 substitutions per base for MinION, compared with 5.8×10^−^^6^ insertions, 9.2×10^−^^6^ deletions and 0.0025 substitutions for Illumina). Three tools [PBcR (Koren *et al.*, 2012), Canu (Berlin *et al.*, 2015) and miniasm (Li, 2016)] were used to assemble MinION pass reads alone, and a fourth [SPAdes (Bankevich *et al.*, 2012)] was used on the combination of MinION pass data and Illumina data to produce a hybrid assembly. PBcR and Canu perform a self-correction step on reads before generating an assembly, whereas miniasm assembles the reads as provided.

All four assemblies had a similar number of contigs and were more contiguous than the assembly using Illumina data alone, with SPAdes producing a single chromosomal contig (Table 1). We ran QUAST (Gurevich *et al.*, 2013) to assess the quality of the assemblies, but found that it could not report all statistics for the miniasm assembly as this fell below the cut-offs for this tool. We used nanopolish (Loman *et al.*, 2015) to correct the miniasm assembly using the raw current signal (pre-base calling) to obtain higher accuracy. The QUAST results showed that the miniasm and nanopolish assembly had a similar number of indels per kb to Canu, although it still had more mismatches per kb (Table 1). Small indels and mismatches were more common in the MinION-only assemblies than the hybrid or Illumina-only assemblies. Assemblies were annotated (Seeman, 2014) and the annotation searched for the housekeeping genes *rpoB* and *hemB* (Hoffmann & Roggenkamp, 2003). These were present in all assemblies with the exception of miniasm, where *hemB* could not be identified. However, the miniasm assembly after nanopolishing had both genes present.

**Fig. 1.**
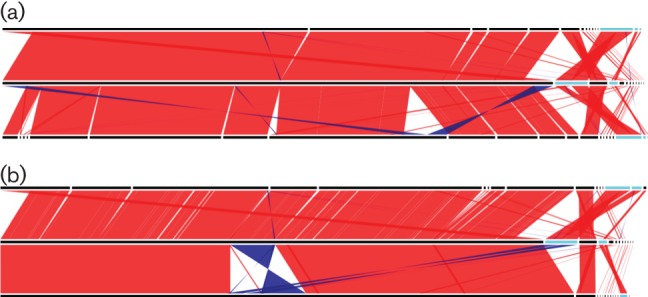
Comparison between (a) Canu (top), manually finished (middle) and PBcR assemblies (bottom) and (b) miniasm and nanopolish (top), manually finished (middle) and SPAdes hybrid assemblies (bottom). Matches are shown where the length of the match is greater than 10 kb or 50 % of the length of the shortest sequence it matches. Forward and reverse matches are colored green and brown, respectively.

The four assemblies were compared to evaluate their ability to reflect the genome structure. A manually finished assembly was produced and used as a reference, from which a single large inversion between the SPAdes assembly and the manually finished assembly was identified (Fig. 1). SPAdes also incorrectly integrated a plasmid into the chromosomal contig, caused by false joins. PBcR made a number of rearrangements compared with Canu (Fig. 1), validating that Canu is an improvement over its predecessor PBcR.

We then evaluated the assembly of all (pass and fail) MinION reads using miniasm and Canu to determine whether adding additional (lower-quality) data would improve the assembly. Adding fail data increased the number of reads by almost 50 % (64 497 versus 43 260) but reduced the mean read length from 5221 bp to 4687 bp. Miniasm run on all reads produced the same number of contigs and a similar mean contig size as when run on pass reads. The longest contig produced with Canu was smaller when using all reads versus pass reads alone (Table S1). With Canu, using pass reads alone led to more reads at the correction step compared with using all reads (35 913 versus 30 728), indicating that working with all reads could cause good-quality data to be discarded during the read correction process. In both cases, using all reads did not produce a single chromosomal contig. We concluded from this that adding fail data did not consistently improve assembly.

We considered the time taken to generate sequence data, together with memory requirements to compute the assembly (Table 1). Almost 50 % of pass reads were generated in the first 6 h, almost 80 % within 9 h and 90 % within 12 h. This gave a theoretical coverage of 20×, 32× and 37×, respectively. Only 31 pass reads were generated in the final 12 h of the 48 h run (<0.1 %). Using pass reads from the first 6 h alone led to a less accurate, fragmented assembly, but subsets of pass reads taken from the first 9 or 12 h of the run generated similar assemblies to pass data from the full 48 h run (Table S1). We also compared speed of data analysis. Miniasm completed assembly within 2 min, but the trade off from using this alone was lower accuracy (Table 1). Nanopolish improved the quality of the miniasm assembly but took over three days to run; Canu took 2 h and produced similar results to the miniasm assembly after nanopolish. With current methods, going from isolate to assembled data in less than 48 h is realistic.

Finally, we evaluated whether these assemblies could be used to identify the presence and position of genes associated with clinically significant drug resistance in the *E. kobei* genome. HiSeq data had detected *bla*_OXA-48_ encoding carbapenem resistance on a 2.5 kb contig and additional antimicrobial-resistance genes in a separate 8.7 kb contig (*sul1, arr, aac3* and *aac6′-IIc*, which encode resistance to sulphonamides, rifampicin and aminoglycosides, respectively), but it was unclear whether these were on the same plasmid, on two different plasmids or chromosomally integrated. All assemblies using MinION data identified the carbapenemase *bla*_OXA-48_ on a contig with plasmid genes. The other resistance genes were identified in proximity to each other on a single large contig along with heavy-metal-resistance genes and plasmid genes. However, the SPAdes assembly misassembled this region into the chromosomal contig (5 Mb). We concluded that there are two separate plasmids carrying resistance determinants of interest.

## Conclusion

MinION data alone could be used with the software described above to generate highly contiguous bacterial assemblies. Canu gave the best results overall, combining low error rate with a highly contiguous assembly. Miniasm created a similar assembly, although the error rate was considerably higher. This means that it has utility in generating an extremely rapid draft answer, but should not be relied upon for high accuracy without additional error correction steps such as nanopolish. SPAdes gave a better accuracy for mismatches and small indels, but created a false join that incorrectly integrated a plasmid into the chromosome. However, SPAdes may be useful where coverage of the genome with MinION data is too low to successfully assemble using MinION data alone. MinION-only assemblies were of sufficient quality to detect and characterise antimicrobial resistance and could be generated rapidly during an outbreak investigation. Whilst other sequencing technologies such as the PacBio RS II generate high-quality long-read sequence data, the portability of the MinION is a potential advantage for medical microbiology.

## Supplementary Data

270 Supplementary File 1

## Supplementary Data

60 Supplementary File 2

## References

[R2] Ashton P. M. Nair S. Dallman T. Rubino S. Rabsch W. Mwaigwisya S. Wain J. O'Grady J.(2015). MinION nanopore sequencing identifies the position and structure of a bacterial antibiotic resistance island. Nat Biotechnol 33 296–300. 10.1038/nbt.3103 25485618

[R3] Bankevich A. Nurk S. Antipov D. Gurevich A. A. Dvorkin M. Kulikov A. S. Lesin V. M. Nikolenko S. I. Pham S.(2012). SPAdes: a new genome assembly algorithm and its applications to single-cell sequencing. J Comput Biol 19 455–477. 10.1089/cmb.2012.0021 22506599 PMC3342519

[R4] Berlin K. Koren S. Chin C. S. Drake J. P. Landolin J. M. Phillippy A. M.(2015). Assembling large genomes with single-molecule sequencing and locality-sensitive hashing. Nat Biotechnol 33 623–630. 10.1038/nbt.3238 26006009

[R6] Boetzer M. Pirovano W.(2012). Toward almost closed genomes with gapfiller. Genome Biol 13 R56. 10.1186/gb-2012-13-6-r56 22731987 PMC3446322

[R5] Boetzer M. Henkel C. V. Jansen H. J. Butler D. Pirovano W.(2011). Scaffolding pre-assembled contigs using SSPACE. Bioinformatics 27 578–579. 10.1093/bioinformatics/btq683 21149342

[R27] Bolger A. M. Lohse M. Usadel B.(2014). Trimmomatic: a flexible trimmer for Illumina sequence data. Bioinformatics 30 2114–2120. 10.1093/bioinformatics/btu170 24695404 PMC4103590

[R7] Bonfield J. K. Whitwham A.(2010). Gap5–editing the billion fragment sequence assembly. Bioinformatics 26 1699–1703. 10.1093/bioinformatics/btq268 20513662 PMC2894512

[R8] Bradley P. Gordon N. C. Walker T. M. Dunn L. Heys S. Huang B. Earle S. Pankhurst L. J. Anson L.(2015). Rapid antibiotic-resistance predictions from genome sequence data for *Staphylococcus aureus* and *Mycobacterium tuberculosis*. Nat Commun 6 10063. 10.1038/ncomms10063 26686880 PMC4703848

[R9] Cao M. Ganesamoorthy D. Elliott A. Zhang H. Cooper M. Coin L.(2015). Streaming algorithms for identification of pathogens and antibiotic resistance potential from real-time MinION sequencing. 10.1186/s13742-016-0137-2 PMC4960868 27457073

[R1] GitHub(2016). *martinghunt/bioinf-scripts*. [online]. https://github.com/martinghunt/bioinf-scripts/blob/master/python/multi_act_cartoon.py. Accessed on 17 May 2016.

[R10] Gurevich A. Saveliev V. Vyahhi N. Tesler G.(2013). QUAST: quality assessment tool for genome assemblies. Bioinformatics 29 1072–1075. 10.1093/bioinformatics/btt086 23422339 PMC3624806

[R11] Hoffmann H. Roggenkamp A.(2003). Population genetics of the nomenspecies *Enterobacter cloacae*. Appl Environ Microbiol 69 5306–5318 . 10.1128/AEM.69.9.5306-5318.2003 12957918 PMC194928

[R12] Hunt M. Silva N. D. Otto T. D. Parkhill J. Keane J. A. Harris S. R.(2015). Circlator: automated circularization of genome assemblies using long sequencing reads. Genome Biol 16 294. 10.1186/s13059-015-0849-0 26714481 PMC4699355

[R13] Judge K. Harris S. R. Reuter S. Parkhill J. Peacock S. J.(2015). Early insights into the potential of the Oxford Nanopore MinION for the detection of antimicrobial resistance genes. J Antimicrob Chemother 70 2775–2778. 10.1093/jac/dkv206 26221019 PMC4566964

[R14] Koren S. Schatz M. C. Walenz B. P. Martin J. Howard J. T. Ganapathy G. Wang Z. Rasko D. A. McCombie W. R.(2012). Hybrid error correction and *De novo* assembly of single-molecule sequencing reads. Nat Biotechnol 30 693–700. 10.1038/nbt.2280 22750884 PMC3707490

[R15] Kurtz S. Phillippy A. Delcher A. L. Smoot M. Shumway M. Antonescu C. Salzberg S. L.(2004). Versatile and open software for comparing large genomes. Genome Biol 5 R12. 10.1186/gb-2004-5-2-r12 14759262 PMC395750

[R17] Li H.(2013). Aligning sequence reads, clone sequences and assembly contigs with BWA-MEM. arXiv:1303.3997v1 [q-bio.GN].

[R28] Li H.(2016). Minimap and miniasm: fast mapping and de novo assembly for noisy long sequences. Bioinformatics 32 2103–2110. 10.1093/bioinformatics/btw152 27153593 PMC4937194

[R16] Li H. Handsaker B. Wysoker A. Fennell T. Ruan J. Homer N. Marth G. Abecasis G. Durbin R. 1000 Genome Project Data Processing Subgroup(2009). The sequence alignment/map format and SAMtools. Bioinformatics 25 2078–2079. 10.1093/bioinformatics/btp352 19505943 PMC2723002

[R18] Loman N. J. Quinlan A. R.(2014). Poretools: a toolkit for analyzing nanopore sequence data. Bioinformatics 30 3399–3401. 10.1093/bioinformatics/btu555 25143291 PMC4296151

[R19] Loman N. J. Quick J. Simpson J. T.(2015). A complete bacterial genome assembled *De novo* using only nanopore sequencing data. Nat Methods 12 733–735. 10.1038/nmeth.3444 26076426

[R20] Otto T. D. Sanders M. Berriman M. Newbold C.(2010). Iterative correction of reference Nucleotides (iCORN) using second generation sequencing technology. Bioinformatics 26 1704–1707. 10.1093/bioinformatics/btq269 20562415 PMC2894513

[R21] Quail M. Smith M. E. Coupland P. Otto T. D. Harris S. R. Connor T. R. Bertoni A. Swerdlow H. P. Gu Y.(2012). A tale of three next generation sequencing platforms: comparison of Ion torrent, Pacific Biosciences and illumina MiSeq sequencers. BMC Genomics 13 341 10.1186/1471-2164-13-341 22827831 PMC3431227

[R29] Quick J. Loman N. J. Duraffour S. Simpson J. T. Severi E. Cowley L. Bore J. A. Koundouno R. Dudas G.(2016). Real-time, portable genome sequencing for Ebola surveillance. Nature 530 228–232. 10.1038/nature16996 26840485 PMC4817224

[R22] Reuter S. Ellington M. J. Cartwright E. J. P. Köser C. U. Török M. E. Gouliouris T. Harris S. R. Brown N. M. Holden M. T. G.(2013). Rapid bacterial whole-genome sequencing to enhance diagnostic and public health microbiology. JAMA Intern Med 173 1397. 10.1001/jamainternmed.2013.7734 23857503 PMC4001082

[R23] Risse J. Thomson M. Patrick S. Blakely G. Koutsovoulos G. Blaxter M. Watson M.(2015). A single chromosome assembly of *Bacteroides fragilis* strain BE1 from Illumina and MinION nanopore sequencing data. Gigascience 4. 10.1186/s13742-015-0101-6 26640692 PMC4670535

[R24] Seemann T.(2014). Prokka: rapid prokaryotic genome annotation. Bioinformatics 30 2068–2069. 10.1093/bioinformatics/btu153 24642063

[R25] Zankari E. Hasman H. Cosentino S. Vestergaard M. Rasmussen S. Lund O. Aarestrup F. Larsen M.(2012). Identification of acquired antimicrobial resistance genes. J Antimicrob Chemother 67 2640–2644. 22782487 10.1093/jac/dks261 PMC3468078

[R26] Zerbino D. R. Birney E.(2008). Velvet: algorithms for *De novo* short read assembly using de Bruijn graphs. Genome Res 18 821–829. 10.1101/gr.074492.107 18349386 PMC2336801

[R31] Zhang J. Kobert K. Flouri T. Stamatakis A.(2014). PEAR: a fast and accurate Illumina Paired-End reAd mergeR. Bioinformatics 30 614–620. 10.1093/bioinformatics/btt593 24142950 PMC3933873

[R30] Zika Real time Sequencing Consortium(2016). http://zibraproject.github.io/.

