# Supplementary material for: Comparison of bacterial genome assembly software for MinION data and their applicability to medical microbiology
Source: Microb Genom. 2016 Sep 8;2(9):e000085. doi: 10.1099/mgen.0.000085 (PMC5320651; doi:10.1099/mgen.0.000085)
Supplement: Supplementary File 1 [file mgen-02-85-s001.docx]

**MGEN-D-16-00047: Judge et al. Comparison of bacterial genome assembly software for MinION data and their applicability to medical microbiology**

**Supplementary Methods: using gap5 to manually finish a genome**


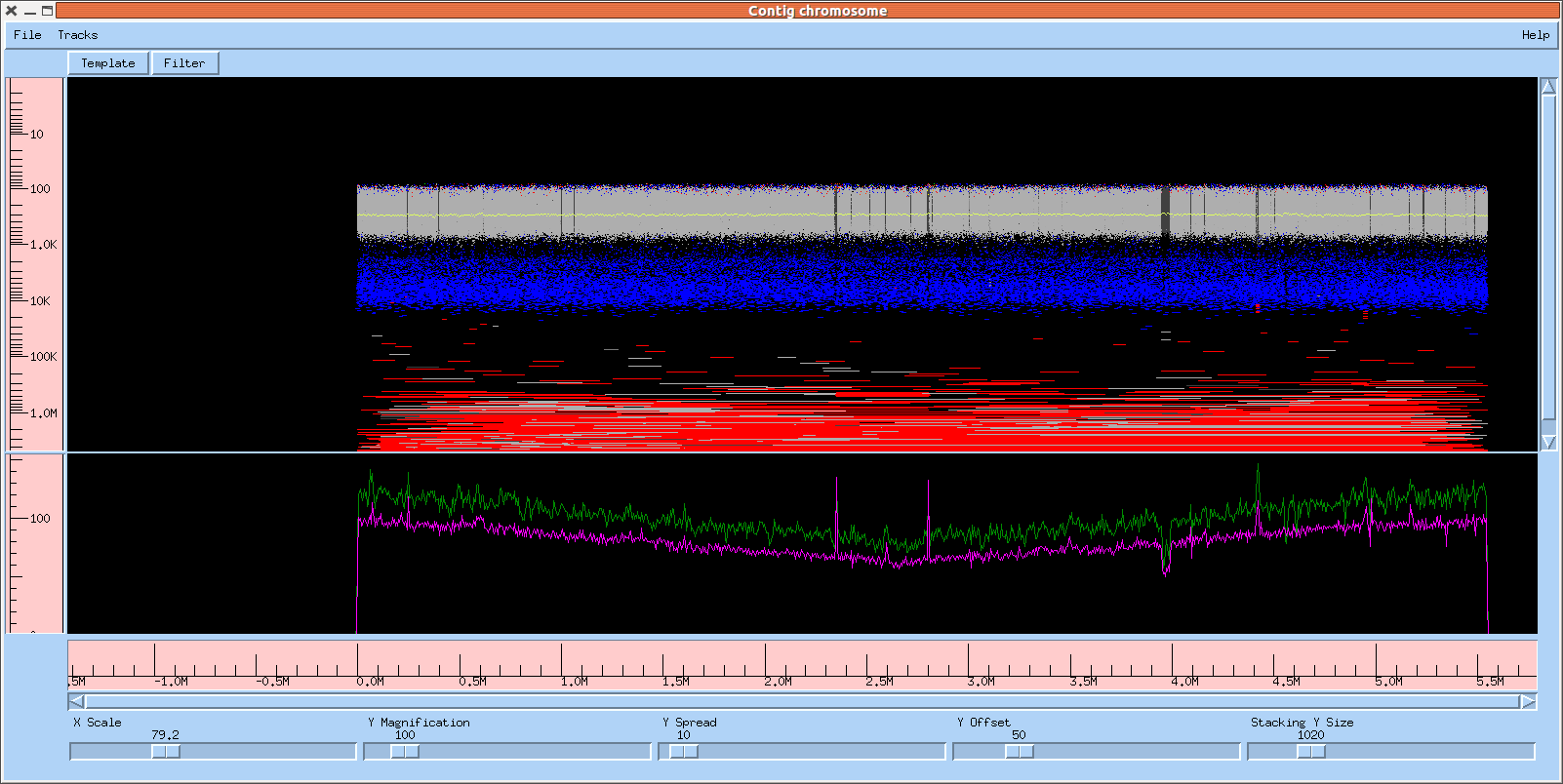


Figure S1. Coverage of the chromosome (orange, top bar) with Illumina reads (grey) and MinION reads after the Canu read correction step (blue). The green line shows coverage by reads, and the purple coverage by fragment (i.e, both reads of an Illumina paired end library). The yellow line in the centre of the grey block shows average fragment size. Illumina read pairs in an unexpected orientation (e.g. both mapping to the forward strand rather than one forward, one reverse) are shown in red. Here, we see the genome evenly covered by both MinION and Illumina reads. The average fragment size (yellow) is level and the distribution of “unexpected orientation” (red) is even, with no spikes that would indicate an assembly error. This indicates that the chromosome is correctly assembled. A “dip” can be seen in the overall coverage of this isolate. This gives further confidence that the assembly of the chromosome is correct. As this is a bacterial chromosome, it is circular and so the contig should be able to be broken at any point. Here, the two ends of the coverage graph would match if joined to form a circle – if the coverage was much higher or lower at the end of the contig than the start it would indicate an error in the assembly.


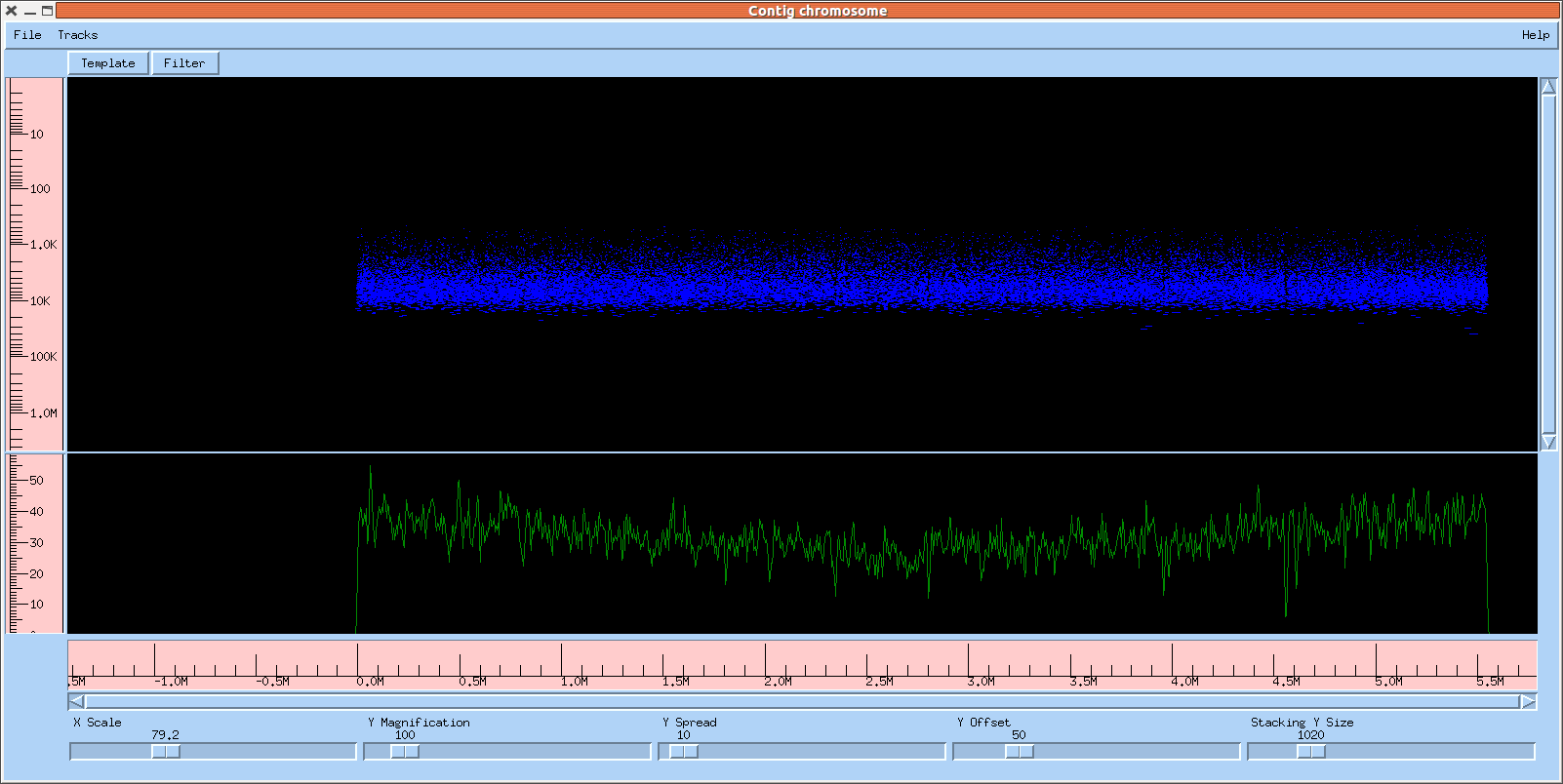


Figure S2. Coverage of the chromosome contig (orange, top bar) with MinION reads after the Canu read correction step (blue).


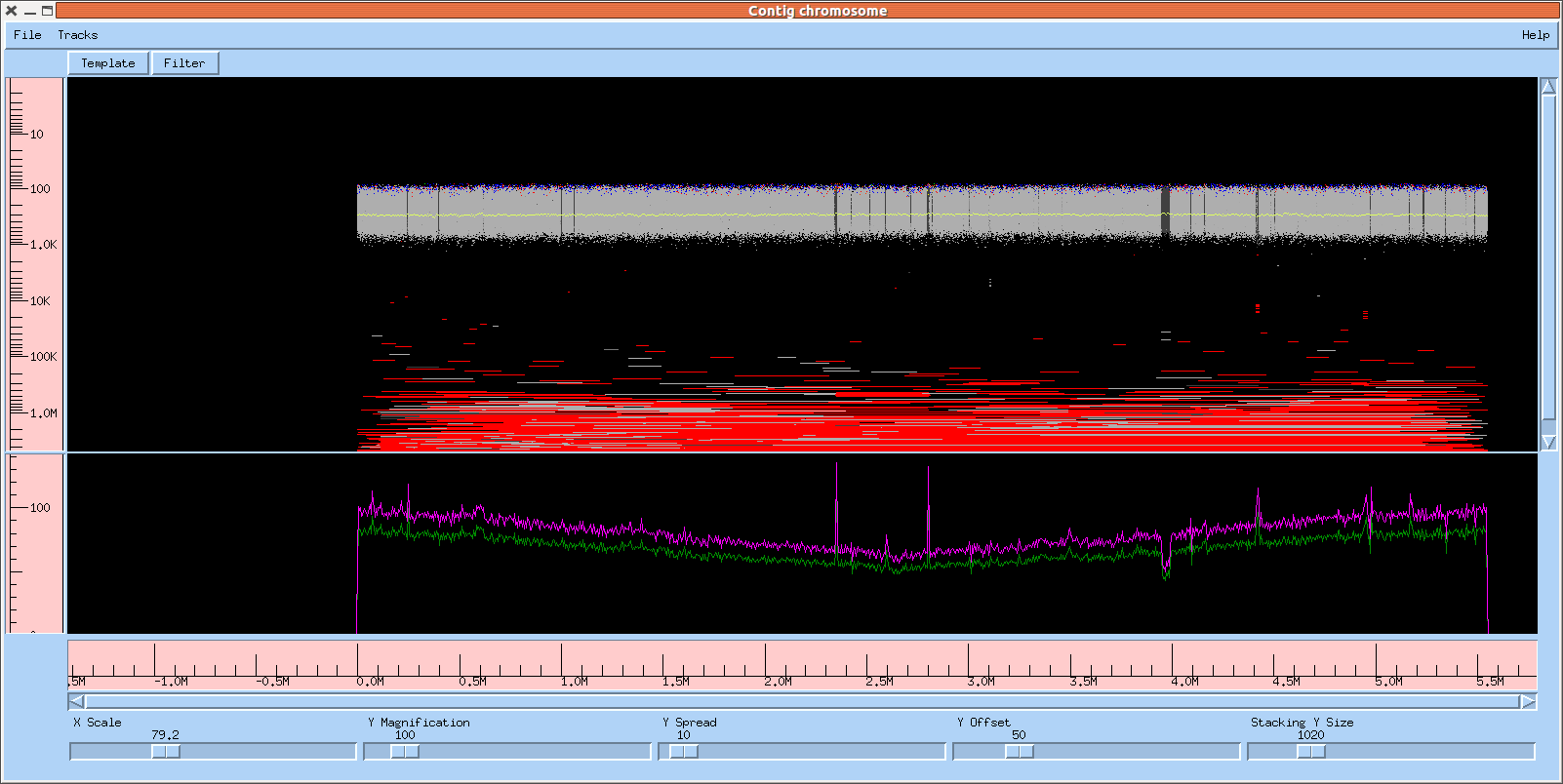


Figure S3. Coverage of the chromosome contig (orange, top bar) with Illumina reads alone (grey).

**Supplementary Text 1: Specification file used to run PBcR**

ovlMemory = 16

ovlStoreMemory= 16000

merylMemory = 16000

ovlThreads = 4

threads=4

merSize=14

falconForce=1

falconOptions=--max_n_read 200 --min_idt 0.50 --output_multi --local_match_count_threshold 0

asmOvlErrorRate = 0.3

asmUtgErrorRate = 0.3

asmCgwErrorRate = 0.3

asmCnsErrorRate = 0.3

asmOBT=0

batOptions=-RS -CS

utgGraphErrorRate = 0.3

utgMergeErrorRate = 0.3
